# Supplementary material for: Protocol for a systematic review of long-term physical sequelae and financial burden of multidrug-resistant and extensively drug-resistant tuberculosis
Source: PLoS One. 2023 May 15;18(5):e0285404. doi: 10.1371/journal.pone.0285404 (PMC10184907; doi:10.1371/journal.pone.0285404)
Supplement: S2 Appendix — (DOCX) [file pone.0285404.s002.docx]

**Identification of studies via other methods**

**Identification of studies via databases and registers**

Records identified from:

Websites (n = ?)

Organisations (n =? )

Citation searching (n =? )

etc.

Records removed *before screening*:

Duplicate records removed (n = ?)

Records marked as ineligible by automation tools (n =? )

Records removed for other reasons (n = ?)

Records identified from*:

Databases (n =? )

Registers (n =? )

**Identification**

Records screened

(n =? )

Records excluded**

(n =? )

Reports not retrieved

(n =? )

Reports sought for retrieval

(n = ?)

Reports sought for retrieval

(n = ?)

Reports not retrieved

(n =? )

**Screening**

Reports assessed for eligibility

(n = ?)

Reports excluded:

Reason 1 (n = ?)

Reason 2 (n = ?)

Reason 3 (n = ?)

etc.

Reports assessed for eligibility

(n = ?)

Reports excluded:

Reason 1 (n =? )

Reason 2 (n = ?)

Reason 3 (n =?)

etc.

Studies included in review

(n = ?)

Reports of included studies

(n = ?)

**Included**

*Consider, if feasible to do so, reporting the number of records identified from each database or register searched (rather than the total number across all databases/registers).

**If automation tools were used, indicate how many records were excluded by a human and how many were excluded by automation tools.

*From:*  Page MJ, McKenzie JE, Bossuyt PM, Boutron I, Hoffmann TC, Mulrow CD, et al. The PRISMA 2020 statement: an updated guideline for reporting systematic reviews. BMJ 2021;372:n71. doi: 10.1136/bmj.n71. For more information, visit: <http://www.prisma-statement.org/>
