# Supplementary material for: Protocol for a systematic review of long-term physical sequelae and financial burden of multidrug-resistant and extensively drug-resistant tuberculosis
Source: PLoS One. 2023 May 15;18(5):e0285404. doi: 10.1371/journal.pone.0285404 (PMC10184907; doi:10.1371/journal.pone.0285404)
Supplement: Attachment — Submitted filename: Response to reviewers.docx [file pone.0285404.s003.docx]

**Point-by-point response**

**Date 4/February/2023**

Jinsoo Min

Academic editor

PLOS ONE

**Subject: Submission of revised manuscript**

Dear Jinsoo Min,

## Thank you for the opportunity to revise our manuscript “**Protocol for a systematic review of long-term physical sequelae and financial burden of multidrug-resistant and extensively drug-resistant tuberculosis**”. We would like to thank the reviewers and the editor for their excellent comments which have substantially contributed to improving our manuscript. We have carefully addressed all the comments improving our responses and the revised manuscript accordingly. Below, we have provided point-by-point responses including how and where the text was modified in the main manuscript. Two separate documents, one with track change and the other a clean version are uploaded to the system. We hope that the revised version is now suitable for publication in PLOS ONE and look forward to hearing from you.

Yours sincerely,

Temesgen Akalu (on behalf of all authors)

| **Editor comments** | **Author’s response** |
| --- | --- |
| Journal Requirements:  When submitting your revision, we need you to address these additional requirements.  1. Please ensure that your manuscript meets PLOS ONE's style requirements, including those for file naming. The PLOS ONE style templates can be found at  <https://journals.plos.org/plosone/s/file?id=wjVg/PLOSOne_formatting_sample_main_body.pdf> and  <https://journals.plos.org/plosone/s/file?id=ba62/PLOSOne_formatting_sample_title_authors_affiliations.pdf> | Thank you, editor, for your valuable comments. Based on your comments the manuscript is updated according to the journal guidelines. |
| 2. Thank you for stating the following financial disclosure:  “No funding”  At this time, please address the following queries:  a)        Please clarify the sources of funding (financial or material support) for your study. List the grants or organizations that supported your study, including funding received from your institution. | KAA is funded by the Australian National Health and Medical Research Council (NHMRC) through an Emerging Leadership Investigator Grant APP1196549. TYA is also supported by Curtin University Higher Degree Research (HDR) Scholarship. We made the change in the online submission and highlighted it in the cover letter. |
| b)        State what role the funders took in the study. If the funders had no role in your study, please state: “The funders had no role in study design, data collection and analysis, decision to publish, or preparation of the manuscript.” | The funders had no role in the study design, data collection, analysis, decision to publish, or preparation of the manuscript. We made the change in the online submission and highlighted it in the cover letter. |
| c)        If any authors received a salary from any of your funders, please state which authors and which funders. | Temesgen Yihunie Akalu (TYA) is a fully funded Ph.D. student at Curtin University, and he is receiving a fortnightly salary until his Ph.D. course is completed (August/2025). Moreover, Dr. Kefyalew Addis Alene (KAA) is a senior researcher at Curtin University and is funded by the Australian National Health and Medical Research Council (NHMRC). He is also receiving a fortnightly salary at Curtin University. However, the other two reviewers (AGB and ACAC) have no salary at Curtin University. |
| d)        If you did not receive any funding for this study, please state: “The authors received no specific funding for this work.” | Based on your comments the source of funding is updated in a point-by-point response above and the online submission system. It is changed and updated as the study is funded by Curtin University. |
| 3. Please include captions for your Supporting Information files at the end of your manuscript, and update any in-text citations to match accordingly. Please see our Supporting Information guidelines for more information: <http://journals.plos.org/plosone/s/supporting-information>. | Based on your fruitful comments, the revision was made accordingly. Hence, a caption for supporting information is added to in-text citations and at the end of the manuscript **(Page 14, lines 318-319)**. |
| **Reviewer 1** | **Author response** |
| **Introduction** | |
| a. Citation number 1 appears to be from the WHO Global TB report, but the 2022 report is available, so the data should be updated. This raises the concern that the numbering of the references does not correspond to the number of the citation in the text, so all citations should be checked to ensure that they correspond to the references. Citation 3 appears before citation 2. The data in the current citation 2 should also be updated with the WHO 2022 report. | Thank you, reviewer, for your valuable comments. According to your comment, we used the WHO 2022 global TB report in the introduction section. The change is also highlighted in the manuscript with track change. Citation 2 is also updated and presented before citation 3. You can see the change on **page 3 lines 44-60**. |
| b. On lines 54 to 59: Are these the three sentences of reference 4? Even so, they do not seem to correspond because reference 4 alludes to data from an American Indian population. | The citation is updated accordingly in the revised manuscript **(Page 3 lines 55 to 60)**. |
| c. In terms of study objectives, meta-analysis is a statistical technique, not a type of study like a systematic review, which cannot always be carried out, depending on the data found in the selected studies. Therefore, meta-analysis should be removed from the objectives. | Based on your comments the phrase meta-analysis is removed. The change is highlighted on **Page 5 lines 92-94**. |
| 2. Methods: |  |
| a. A 2015 version of PRISMA is cited, but should be replaced by the latest version in 2020. | Thank you, reviewer, for your suggestion. As you said we are planning to use PRISMA-20 for the systematic review and meta-analysis when the review process is completed. However, for protocols, we use PRISMA-P instead of PRISMA. As far as our search is concerned, we couldn’t find PRISMA-20 for protocol. The available PRISMA-20 is not for protocols, but it is for reporting findings of systematic reviews and meta-analyses after the plan is completed. Therefore, the most updated reporting item for systematic review and meta-analysis protocols is the PRISMA-15. Based on recommendations we will use PRISMA -20 for the results once the study is completed. However, we authors are keen to welcome you if you come up with an updated PRISMA-P version 20. |
| b. Within the Search Strategy, Central (trials) from the Cochrane Library and other grey literature sources (Medicines/Drug Agencies). For strategy number 3 (Table 1), it is recommended to introduce more search terms, such as: ("Cost of Illness" OR "Cost of Sickness" OR "Financial Stresses” OR "Financial Stress" OR "financial pressure*" OR "Financial Hardship" OR "Financial Hardships" OR "Financial Challenges" OR "Economic Burden" OR "Financial toxicity" OR "Financial Impact" OR "health economics" OR "Financial Distress"). | Based on your valuable comments, an additional search from the Cochrane Central Register of Controlled Trials (CENTRAL trial) and Gray literature (Medicines/Drug Agencies) will be made **(Page 5 lines 102-105)**. Moreover, the search term for strategy number 3 is updated accordingly. The change is highlighted in **table 1 (searching strategy)**. |
| c. Within the Quality Assessment section, the Cochrane Handbook for Systematic Reviews of Interventions (Version 6.3, 2022) should be incorporated to assess the risk of bias in randomised and non-randomised studies of interventions. In addition, the Newcastle-Ottawa Scale (NOS), the Meta-analysis Of Observational Studies in Epidemiology (MOOSE) statement and the Grading of Recommendation Assessment, Development, and Evaluation (GRADE) need references. | It is updated in the revised manuscript and modified as “*The Cochrane Handbook for Systematic Reviews of Interventions (Version 6.3, 2022) will be used for assessing* the *quality of experimental studies*”. Besides, we also added references to the Meta-analysis of Observational Studies in Epidemiology (MOOSE) statement and the Grading of Recommendation Assessment, Development, and Evaluation (Grade). The change is highlighted on pages **9-10 lines 173-183**. |
| 3. Related to Data Availability: As this is a systematic review protocol, no data is currently available in an open access repository, but it would be advisable that the search strategies launched in the different bibliographic databases, as well as the RIS files of the retrieved documents, could be made available once the protocol has been executed. | As you said no data is available in an open-access repository as it is a protocol. This is why we authors didn’t mention the data availability statement in the manuscript. However, once the protocol has been executed the data will be available in RIS files of the retrieved documents, in proposed search strategies of different databases, and references of included studies will be cited in the manuscript. As a result, the data will be easily available online to everyone interested. So, the availability statement for protocols is not applicable at the movement. |
| **Reviewer 2** | **Author’s Response** |
| Line 46-48: I recommend using the recent (2022) global TB report for reporting some figures such as the global and local burden of TB. Almost all the estimates in the introduction should be updated using recent reports. | Based on your fruitful comment updated world health organization (WHO) 2022 report was used throughout the introduction section. So, the change is highlighted in a manuscript with a track change. It is also highlighted on **page 3 lines 44-60.** |
| - In the first search term, I recommend including “Totally drug-resistant tuberculosis” as one of the search terms | Based on your comment the search strategy is updated in the revised manuscript. Hence, totally drug-resistant tuberculosis (TDR) is included in the revised manuscript **(Table 1)**. |
| - In the third search term, financial sequel, it is better to add “catastrophic health expenditure”, as one of the search terms | Similarly, the third search term is updated and the phrase “catastrophic health expenditure” is included in the revised version. The revision is highlighted in **(Table 1)**. |
| - In the fourth search terms, I recommend including terms such as “sequels” and “effect” | The term “sequels” and “effect” are incorporated in the fourth search term based on your recommendations **(Table 1)**. |
