# Supplementary material for: Protocol for a systematic review of long-term physical sequelae and financial burden of multidrug-resistant and extensively drug-resistant tuberculosis
Source: PLoS One. 2023 May 15;18(5):e0285404. doi: 10.1371/journal.pone.0285404 (PMC10184907; doi:10.1371/journal.pone.0285404)
Supplement: Attachment — Submitted filename: Point-by-point response.docx [file pone.0285404.s004.docx]

**Point-by-point response**

Date 30/03/2023

Jinsoo Min

Academic Editor, PLOS ONE

**Subject: Submission of revised manuscript**

Dear Jinsoo Min,

Thank you for providing an additional opportunity to revise our manuscript “**Protocol for a systematic review of long-term physical sequelae and financial burden of multidrug-resistant and extensively drug-resistant tuberculosis”**. We are grateful for the reviewers' and editor’s comments and believe that the revised manuscript is now stronger because of their feedback. We have carefully addressed all the comments provided by the editor and reviewers in our responses and incorporated related changes into the manuscript. Below, we have provided point-by-point responses including how and where the text was modified in the main manuscript. Two separate documents, one with track change and the other a clean version are uploaded to the system. We hope that the revised version is now suitable for publication in PLOS ONE and look forward to hearing from you in due course.

Yours sincerely,

Temesgen Akalu (on behalf of all authors)

| **Editor comments** | **Author response** |
| --- | --- |
| Introduction section is too long. Please, modify the introduction section and reduce to two or three paragraphs. Remnant contents could be moved to the discussion section. | The introduction section is now modified and reduced into three paragraphs **(pages 3 to 4, lines 46-67)**. Some of the remaining sections moved to the discussion section as suggested by the editor on **pages 9-10, lines 176-199.** |
| Please, summarize the "methods and materials" and insert a new paragraph immediately after the "Discussion" heading. Write a new paragraph of 3-4 sentences at the end of the manuscript summarizing all the manuscript as a conclusion. | Following the journal requirements and the editor’s suggestion, we have now summarized the methods and material sections and provided a brief conclusion after the discussion sections **(Page 10, lines 201-205)**. |
| Try to avoid unnecessary use of the abbreviation, which was only used for once or twice. page 3 line 45 : COVID-19 page 4 line 84 : DS page 11 line 205 : COPD | All unnecessary use of abbreviations is now modified in the revised version of the manuscript. |
| Insert the full term when the abbreviation was first used in the manuscript. page 2 line 30: RR page 3 line 59 : DR-TB page 11 line 221 : PRISMA ?? -> this abbreviation was used elsewhere in the manuscript before. | All abbreviations are corrected in the revised manuscript. |
| others page 9 line 164 : change "diabetes" to "Diabetes" (Use capital letter) page 11 line 215 : Please, delete sub-heading, "strengths and limitations". | The sub-heading is removed, and the term “diabetes” is changed to “Diabetes”. |
| Please, check and correct the all the grammatical errors.  page 5 line 93 : aim -> aims | All grammatical errors are now corrected in the revised manuscript. |
| **Reviewer 1 comment** | **Author response** |
| Reviewer #1: Although the authors use PRISMA-P (2015) for the systematic review protocol reporting items, for the supplementary file (S1 Table: Preferred Reporting Items for Systematic Reviews and Meta-analyses (PRISMA) flow diagram for the summary of systematic review study selection process) they should use PRISMA 2020 (flow diagram for new systematic reviews which included searches of databases, registers and other sources (<http://www.prisma-statement.org/PRISMAStatement/FlowDiagram>)) | Thank you for your valuable comment. In the revised version of the manuscript, PRISMA-P (2015) is changed by PRISMA-P (2020). |
